# Combined using of paclitaxel and salinomycin active targeting nanostructured lipid carriers against non-small cell lung cancer and cancer stem cells

**DOI:** 10.1080/10717544.2019.1580799

**Published:** 2019-03-16

**Authors:** Jianwen Zhou, Mingshuang Sun, Shanshan Jin, Li Fan, Wenquan Zhu, Xiaoyu Sui, Lixin Cao, Chunrong Yang, Cuiyan Han

**Affiliations:** a School of Pharmacy, Jiamusi Medical University, Jiamusi, China;; b School of Pharmacy, Qiqihar Medical University, Qiqihar, China;; c School of Pharmacy, Taishan Medical University, Taian, China;; d Department of Pharmaceutical Preparations, Beijing Dezhong Wanquan Medicine Technology Co., Ltd, Beijing, China;; e Research Institute of Medicine & Pharmacy, Qiqihar Medical University, Qiqihar, China;; f Department of Orthopedics, the First Affiliated Hospital of Qiqihar Medical University, Qiqihar, China

**Keywords:** Nanostructured lipid carriers, salinomycin, paclitaxel, combined therapy, active targeting

## Abstract

The existing of avidity cancer stem cells (CSCs) made it an optical strategy to kill cancer cells and CSCs at the same time. Here, we constructed a CSCs specific nanocarrier naming T-S-NLC using the CD133+ targeting peptide TISWPPR (TR) as the targeting moiety attached to the distal end of PEG on salinomycin (Sal) loaded nanostructured lipid carriers (NLC), its pharmaceutical characteristics proved it 128.73 ± 2.09 nm, anionic spheroid with sustained release profile. It's *in vitro* targeting effect in CD133+ CSCs indicated that it exhibited superior CSCs internalization over non-modified NLC or free drug. Afterwards, it was used in combination with previously designed EGFR specific A-P-NLC (AEYLR peptide-PEG-modified paclitaxel loaded NLC) to achieve the goal to kill the cancer cells and CSCs, simultaneously. The *in vitro* tumor targeting effect of T-S-NLC + A-P-NLC was affirmed by cellular uptake and proliferation inhibition effect in NCI-H1299 and S180 cell lines showing advanced results over single preparation groups. *In vivo* tumor targeting effect in S180 tumor-bearing mice also validated the better tumor accumulative effect of the combined group. Last but not least, the *in vivo* antitumor effect strongly identified the greater tumor suppression effect of T-S-NLC + A-P-NLC than single preparation groups or combined use of free drugs while maintaining a good living state of the mice. To sum up, the combined usage of PTX and Sal active targeting NLC naming A-P-NLC + T-S-NLC which killed cancer cells and CSCs at the same time was a promising drug delivery system.

## 1. Introduction

Lung cancer is one of the most common cancers, which is the leading cause of cancer-related death among various cancers worldwide (Torre et al., 2016; Mao et al., 2016). Non-small-cell lung cancer (NSCLC) is the predominant subgroup of lung cancer with a high opportunity of recurrence and metastasis (Ettinger et al., 2017; Siegel, et al., 2018). Paclitaxel (PTX), as the first line drug in treating NSCLC, has achieved good antitumor effect in the past (Francis et al., 1995; Hájek, 1996). However, due to its poor water solubility, PTX needs a vast amount of Cremophor EL and ethanol to be dissolved when used in clinic, which two could possibly arouse severe allergy, meanwhile, the lack of cancer cell specificity of PTX could also cause adverse effect like myelosuppression, neurotoxicity, cardiovascular toxicity, gastrointestinal reaction, and hair loss (Sparreboom et al., 1999; Gelderblom et al., 2001; van Zuylen et al., 2001).

To improve the cancer cell specificity and alleviate the side effects of small molecular chemotherapy drugs like PTX, numerous papers have been published in the field of nano drug delivery carriers, of which the superior antitumor effect has been validated (Shin et al., 2012; Werner et al., 2013; Chen et al., 2014; Pandey et al., 2015; Fu et al., 2016; Giordano et al., 2017; Zhang et al., 2018). Nevertheless, it has been mentioned that the drug resistance of cancer stem cells (CSCs) could cause the cancer recurrence and metastasis, which leads to the failure of the chemotherapy (Dallas et al., 2009; Heery et al., 2017). In this case, it seems that the optimal plan to confront cancer is to kill the cancer cells and the CSCs at the same time. Salinomycin (Sal) is a CSCs specific drug discovered in recent years (Gupta et al., 2009; Naujokat & Steinhart, 2012). Researches have revealed its definite therapeutic effect against lung CSCs (Arafat et al., 2013). Herein, it seemed that the combination of PTX and Sal to treat NSCLC could kill the cancer cells and the CSCs at the same time (Larzabal et al., 2013). However, as a small molecule drug, Sal often exhibited severe adverse effects like acute renal failure and even death (Story & Doube, 2004; Zhang et al., 2009; Li et al., 2010). Thus the key problem of the combination of the two drugs is how to reduce the adverse effects.

The ideal strategy is to target PTX to the lung cancer cells and Sal to the CSCs. The previously designed small peptide AEYLR (AR) could specifically recognize the over-expressed epidermal growth factor receptor (EGFR) on the lung cancer cell membrane, AR conjugated nanostructured lipid carriers (NLC) was also proved to target the EGFR overexpressed cells and increase the drug accumulation inside the cancer cells (Han et al., 2013, 2014). In this manuscript, we use the same method to deliver PTX to cancer cells: NLC is the PTX carrier with the surface modified with PEG and AR at the distal end of PEG (A-P-NLC) to achieve the active targeting effect towards the EGFR overexpressed NCI-H1299 cells (Han et al., 2013; Liao et al., 2015). For the purpose of targeting the CSCs, we employ the TISWPPR (TR) peptide to the distal end of the PEG on the Sal loaded NLC surface to construct T-S-NLC. Since TR peptide could recognize and bind to CD133+ (Tian et al., 2011) which is a landmark on the membrane of the NCI-H1299 stem cells, the designed T-S-NLC could theoretically target to the NCI-H1299 stem cells. And with the combination of A-P-NLC and T-S-NLC, we could possibly kill the cancer cells and CSCs, avoid the cancer recurrence and metastasis, improve the anticancer effect and lower the adverse effect at the same time.

## 2. Materials and methods

### 2.1 Materials, cell line, and animals

Compritol 888 ATO, ATO-5 were kindly donated by Gattefossé Co., Ltd., France. Glyceryl Monostearate (GMS) was purchased from KLK oleochemical industry Co., Ltd., Malaysia. Medium-chain triglyceride (MCT) was purchased from Fenglijingqiu commercial and Trading Co., Ltd., China. DSPE-PEG2000-TR with greater than 98% purity was synthesized by TeraBio Technology Co., Ltd., China. PTX was purchased from Menster Biotec Co., Ltd., China. Self-made EGFR specific small peptide AR-PEG- modified PTX loaded NLC (A-P-NLC) with an average size of 43.92 ± 0.76 nm and drug loading efficiency of 1.97 ± 0.25%. Sal was purchased from Cayman Chemical Co., Ltd., USA. Kolliphor ELP, Solutol HS15, and Kolliphor EL were all generously presented by BASF Co., Ltd., Germany. Polyoxyethylene (40) stearate (P40) was purchased from Sigma reagent company, USA. Coumarin-6 (Cou 6) and Dir as the fluorescent dye were purchased from Ruitaibio Company, China. Self-made AR-PEG- modified Cou 6 and Dir loaded NLC (A-Cou6-NLC and A-Dir-NLC) showed average size of 50.22 ± 1.93 nm, 47.51 ± 0.84 nm and drug loading efficiency of 1.65 ± 0.39%,1.27 ± 0.59%, respectively. RPMI 1640 culture medium and fetal bovine serum were both purchased from Gibco Co., Ltd., USA.

Human lung cancer cell line NCI-H1299 and mouse sarcoma cell line S180 were purchased from the Cell Bank of Chinese Academy of Sciences, and cultured in the RPMI 1640 culture medium with 19% fetal bovine serum.

KM mouse with half male and half female were purchased from the Experimental Animal Department of Harbin Medical University. Our experiments that concerned animals all strictly followed the National Institutes of Health guide for the care and use of Laboratory Animals (NIH Publications No. 8023, revised 1978).

### 2.2 Methods

#### 2.2.1. Preparation and pharmaceutical characteristics of various NLC

Melt emulsification and solidation method were employed to construct various kinds of NLC. Briefly, ATO-5, MCT, Solutal HS15, Kolliphor EL were mixed and melted at 80 °C to get the transparent oil phase, P40 was dispersed in distilled water at the same temperature to get the aqueous phase and added into the oil phase dropwise, the emulsion was cooled at 4 °C to obtain the NLC after the further emulsification of the mixture for 10 min to get the Sal, Cou6 or Dir loaded NLC naming S-NLC, Cou6-NLC or Dir-NLC, Sal, Cou6 or Dir was added to the oil phase of the preparation during the procedure. To modify the S-NLC or Cou6-NLC or Dir-NLC with TR, DSPE-PEG2000-TR was added to to the oil phase, and the corresponding name of the preparation was T-S-NLC, T-Cou6-NLC or T-Dir-NLC.

To know the size and zeta potential of T-S-NLC, T-Cou6-NLC, and T-Dir-NLC, the preparation was diluted 50 times by distilled water and measured by Malvern Zetasizer ZS (Malvern, Worcestershire, UK), the test was conducted for three times.

To learn the entrapment efficiency (EE%) and drug loading (DL%) of T-S-NLC, ultrafiltration centrifugation method was used. Briefly, T-S-NLC was placed into the inner tube of the ultrafiltration centrifugation tube and centrifuged at 10,000 rpm for 20 min, then the Sal content in the centrifugate representing the free drug content was tested using HPLC. The DL% of T-Cou6-NLC and T-Dir-NLC were disposed by the same method above and measured by fluorescence spectrophotometry. EE% and DL% were calculated by Equation 1 and Equation 2, with *W*_A_, *W*_F_ and *W*_TL_ represent the added Sal weight, free Sal weight, and total lipid weight.

(1) $$\mathrm{EE}\%=\frac{W_{A}-W_{F}}{W_{A}}$$

(2) $$\mathrm{DL}\%=\frac{W_{A}-W_{F}}{W_{\mathrm{TL}}}$$

One milliliter of T-S-NLC was placed in a dialysis bag (MW 8–14 KDa) with both ends sealed and immersed into 50 mL drug release medium (the release medium was pH 7.4 PBS with 20% ethanol and 6% SDS) and shaken at 37 °C at 200 rpm to study the drug release behavior. An aliquot of 1 mL release medium was withdrawn at a predicted point and a supplement of the same temperature was added immediately. The withdrawn release medium was evaporated in the rotary evaporators and re-dissolved in 50% methanol, and the Sal content was tested by HPLC. After the drug release study, the accumulative drug release profile was calculated.

#### 2.2.2. *In vitro* targeting effect of T-S-NLC to NCI-H1299 CSCs

##### 2.2.2.1. Separation and identification of CD133+ CSCs

The regularly cultured NCI-H1299 cells were trypsinized into single cells using 0.25% trypsin and harvested. Then the cells were washed with the separation buffer and centrifuged at 500 rpm for 3 min, the cell pellet at the bottom of the tube was mixed with 10 μL CD133+ immunomagnetic beads and incubated in dark place at 4 °C for 30 min. Afterwards, the cells were washed with PBS for 3 times and resuspended in 1 mL separation buffer. The cell suspension was added to the premoistened separation column, and washed out with 1.5 mL PBS with the aid of the assistor, the eluant which contained the CD133+ CSCs was collected. (Shen et al., 2018)

To identify the CD133+ CSCs, the cells collected above were washed with PBS, then cocultured with PE-labeled CD133+ antibody at 4 °C for 30 min, centrifuged at 500 rpm for 5 min to remove the unbonded antibody, resuspended with PBS, transferred to the flow cytometry tube and detected at the flow cytometry.

##### 2.2.2.2. *In vitro* CSCs targeting assay of T-S-NLC

CD133+ CSCs were seeded at the laser confocal microscopy special plate at the density of 1 × 10^6^ cells/well and incubated overnight. At the second day, the cell culture was replaced with a different solution including Cou6, Cou6-NLC, and T-Cou6-NLC dispersed in the RPMI culture medium, the Cou6 concentration was 50 ng/ml in each group. After the further 3 h incubation, each solution was abandoned and the cells were rinsed with cold PBS and stained with 0.5 μg/ml Hoechst 33258 for 20 min, fixed with 4% paraformaldehyde for 15 min, and visualized under the laser confocal microscopy.

##### 2.2.2.3. Cell proliferation inhibition test

CD133+ CSCs were seeded in the 96-well plate at the density of 1 × 10^4^ cells/well and incubated overnight. At the second day, Sal, S-NLC, and T-S-NLC were diluted by complete culture medium which was used to replace the original culture medium at the Sal concentration of 0.53 μg/ml and incubated for another 48 h, then 10 μL CCK-8 solution was added into each well and another 4 h incubation was conducted, then the absorbance of each well was read at 450 nm using the microplate reader (Guo et al., 2018). The cell proliferation inhibition rate (PI%) was calculated using Equation 3 (*A*_t_ represented the absorbance of test groups including Sal, S-NLC, and T-S-NLC, *A*_B_ represented the blank group in which the wells were filled with only culture medium and no cells, and *A*_c_ represented the controlled group in which the wells were filled with cells and culture medium).

(3) $$\mathrm{PI}\left(\%\right)=\frac{\left(A_{t}-A_{B}\right)}{\left(A_{c}-A_{B}\right)}\times 100\%$$

#### 2.2.3. Combination effect of A-P-NLC and T-S-NLC

After the confirmation of the CD133+ targeting effect of T-S-NLC, it was used in combination with previously designed A-P-NLC, of which the EGFR targeting effect was proved (Sun et al., 2018).

##### 2.2.3.1. *In vitro* qualitative targeting assay

To investigate the superiority of the combination of A-P-NLC and T-S-NLC *in vitro*, we conducted the cellular uptake assay of Cou6, T-Cou6-NLC, A-Cou6-NLC, and T-Cou6-NLC + A-Cou6-NLC in EGFR positive NCI-H1299 and S180 cells (Wang et al., 2012) (with the Cou6 concentration at 50 ng/ml in Cou6, T-Cou6-NLC, A-Cou6-NLC groups and 25 ng/ml in T-Cou6-NLC + A-Cou6-NLC group). The cells were seeded in the laser confocal microscopy special dish at the density of 1 × 10^6^ cells/well and incubated overnight. At the second day, the culture medium was replaced by RPMI1640 culture medium containing Cou6, A-Cou6-NLC, T-Cou6-NLC, and A-Cou6-NLC + T-Cou6-NLC at predicted Cou6 concentration and incubated for another 3 h, then the cells were washed with cold PBS for 3 times, nucleic stained by Hoechst 33258 for 20 min, fixed with 4% paraformaldehyde and observed with the laser confocal microscopy.

##### 2.2.3.2. *In vitro* quantitative assay

The cells were seeded in 24-well plate at the density of 1 × 10^6^ cells/well and incubated overnight. At the second day, the cell culture medium was replaced by incomplete RPMI 1640 culture medium containing Cou6, A-Cou6-NLC, T-Cou6-NLC, and A-Cou6-NLC + T-Cou6-NLC at the same Cou6 concentration above and incubated for another 3 h, then the cells were washed for 3 times with cold PBS, trypsinized, resuspended in PBS and tested by flow cytometry to learn the fluorescence intensity.

##### 2.2.3.3. Cell proliferation inhibition effect assay

Combined drug groups of PTX + Sal, P-NLC + S-NLC, A-P-NLC + T-S-NLC with the PTX and Sal concentration ratio at 1:1, and single drug groups of A-P-NLC and T-S-NLC were used to test the proliferation inhibition effect of NCI-H1299 and S-180 cells.

The cells were seeded in the 96-well plate at the density of 1 × 10^4^ cells/well and incubated overnight. At the second day, each group of preparation was diluted by complete culture medium and employed to replace the original culture medium at the drug concentration of 0.53 μg/ml and incubated for another 48 h, then 10 μL CCK-8 solution was added into each well and another 4 h incubation was conducted, then the absorbance of each well was read at 450 nm using the microplate reader. The NCI-H1299 and S180 cell proliferation inhibition rate was calculated using Equation 3, separately.

#### 2.2.4 *In vivo* tumor targeting effect of A-P-NLC + T-S-NLC

A-Dir-NLC, T-Dir-NLC, and A-Dir-NLC + T-Dir-NLC were used to study the *in vivo* tumor targeting effect. S180 tumor-bearing mice were randomly divided into 3 groups with 6 mice in each group after the tumor volume got 200 mm^3^. A-Dir-NLC, T-Dir-NLC, and A-Dir-NLC + T-Dir-NLC were injected through the tail vein to the mice in corresponding group at the Dir amount of 2.5 mg/kg, the mice were shaved, anesthetized, and photographed at 1 h, 3 h, 5 h, and 7 h. Twenty-four hours later, the mice in each group were sacrificed and the tumor, heart, liver, spleen, lung, and kidney were taken out and photographed. The *in vivo* tumor targeting effects of different preparations were analyzed according to the above pictures.

#### 2.2.5. *In vivo* antitumor effect of combined T-S-NLC + A-P-NLC

S180 ascites tumor cells were planted to the left armpit of the mice, the mice were randomly divided into 10 groups with 6 mice in each group after the tumor volume got 200 mm^3^. Saline, Sal, PTX, Sal + PTX, S-NLC, P-NLC, S-NLC + P-NLC, T-S-NLC, A-P-NLC, and T-S-NLC + A-P-NLC were injected to the corresponding mice at the drug dose of 5 mg/kg through the tail vein, every other day for 7 times. The living state, body weight, and tumor volume (V = AB^2^/2, A represent the length of the tumor and B represent the width) of the mice in each group were documented every other day. The mice were sacrificed 24 h after the final dose, the tumors were peeled off and weighed, and the tumor inhibition rate (IR%) was calculated with Equation 4, in which W*_d_* meant the tumor weight in drug groups and W*_s_* meant the tumor weight in Saline group.

(4) $$\mathrm{IR}\%=\left(1-\frac{W_{d}}{W_{s}}\right)\times 100\%$$

## 3. Results and discussion

### 3.1. Pharmaceutical characteristics of various NLC

**Figure 1.**
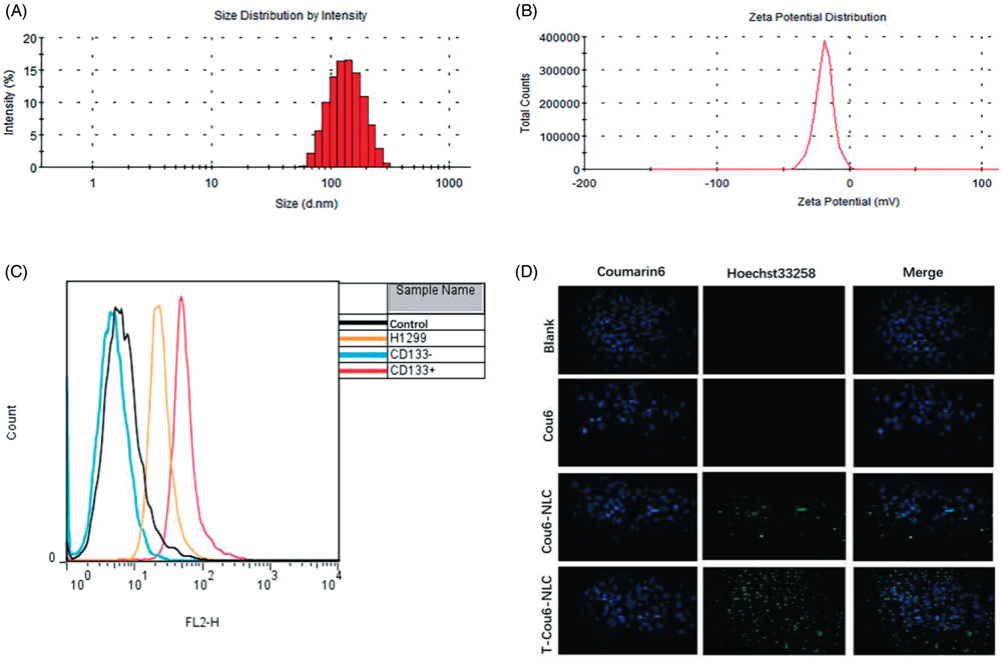
The size distribution (A) and zeta potential distribution (B) of T-S-NLC. The flow cytometry result of CD133+ NCI-H1299 CSCs separated using the immunomagnetic beads (C). *In vitro* cellular uptake study of various preparations in CD133+ CSCs observed by the laser confocal microscopy (D). The green signal represented Cou6 and the blue signal represented the Hoechst 33258.

The size of T-Cou6-NLC, T-Dir-NLC, and T-S-NLC were 129.51 ± 0.44 nm, 125.63 ± 1.75 nm, and 128.73 ± 2.09 nm (Figure 1(A)), indicating that the T-Cou6-NLC, T-Dir-NLC, and T-S-NLC met the demand size range of tumor enhanced permeability and retention effect (EPR) which was the golden rule of the passive tumor targeting effect. The zeta potential of T-S-NLC was −28.3 ± 0.4 mv (Figure 1(B)), suggesting its good physical stability. The EE% and DL% of T-S-NLC were 95.62 ± 1.46 and 1.02 ± 0.06%, the DL% of T-Cou6-NLC and T-Dir-NLC were 1.09 ± 0.15% and 1.15 ± 0.09% indicating the satisfactory drug loading capacity of the carrier. The drug accumulative release was above 95% in 24 h, and the Sal released from the carrier in a fast-slow pattern, which is probably because the initial drug release was due to the desorption of the Sal from the carrier surface, and the later was the Sal released from the T-S-NLC. The overall sustained drug release behavior could maintain that the drug released after the carrier got to the tumor area by EPR effect and reduce the drug leakage during the circulation, greatly decreased the occurring of the adverse effect to normal tissues.

### 3.2. *In vitro* targeting assay of TR modified NLC to CD133+ NCI-H1299 CSCs

The CD133+ NCI-H1299 CSCs were separated from regularly cultured cells using the immunomagnetic beads and identified by flow cytometry. The result is shown in Figure 1(C), the original NCI-H1299 cells contained 3.69% CD133+ CSCs, and the ratio increased to 75.35% after mixed with the immunomagnetic beads and separated with the magnetic column. The eluant of the cells without mixing with the immunomagnetic beads gave nearly no CD133+ CSCs, indicating the feasibility of the CSCs separating method.

The separated CD133+ CSCs were employed to test the *in vitro* targeting effect of the TR modified NLC by the method of laser confocal microscopy, and the result is shown in Figure 1(D). Compared with the blank group, Cou6 solution showed nearly no cellular uptake however, after loading into NLC (Cou6-NLC), its green signal appeared within the cells, suggesting that the Cou6 was uptaken with the aid of the drug carrier. After the modification of NLC surface with CD133+ targeting peptide TR (T-Cou6-NLC), the green signal of Cou6 got significantly stronger due to the specific binding of TR peptide to the CD133+ on the CSCs membrane, these results illustrated that TR modified NLC could enhance the drug internalization efficiency of CD133+ CSCs to a great extent.

### 3.3. Cell proliferation inhibition test of T-S-NLC to CD133+ NCI-H1299 CSCs

To further confirm the cell proliferation inhibition effect of Sal and Sal preparations, we conducted the corresponding assay using CCK-8 method. After co-incubated with CD133+ CSCs for 48 h, Sal, S-NLC, and T-S-NLC showed various cell proliferation inhibition effects. The inhibition ratio of Sal, S-NLC, and T-S-NLC were 17.06 ± 2.10%, 34.14 ± 2.42%, and 68.58 ± 1.46%, revealing that the cell toxicity of Sal was enhanced after loading into the NLC (*p* < .01), and further increased after the modification of CD133+ targeting peptide TR to the NLC (*p* < .01), the results showed similar trends with the cellular uptake assay and affirmed the specific interaction between TR peptide and CD133+.

### 3.4. *In vivo* tumor targeting effect of the combination of AR modified NLC and TR modified NLC

CSCs exist in malignant tumors and take major responsibility for the recurrence and metastasis of cancers. Thus, the strategy of killing cancer cells and CSCs at the same time seemed to be an ideal idea. In the present manuscript, we employ the NSCLC cells NCI-H1299 as the model cells to validate the antitumor effect of our drug delivery system which actively targets to the cancer cells and the CSCs. Since EGFR was overexpressed on the membrane of NCI-H1299 and S180 cells, and CD133+ was a marker of CSCs, these two components were used as the target spots of our drug delivery system.

In our early publications, the small peptide AR has been proved to be EGFR specific, and AR modified NLC (A-NLC) could also actively target the EGFR overexpressed cells. The CSCs targeting effect of TR modified NLC (T-NLC) was proved in above assays. In this section, we combined A-NLC and T-NLC to treat the NCI-H1299 and S180 cells to testify the advanced antitumor effect.

**Figure 2.**
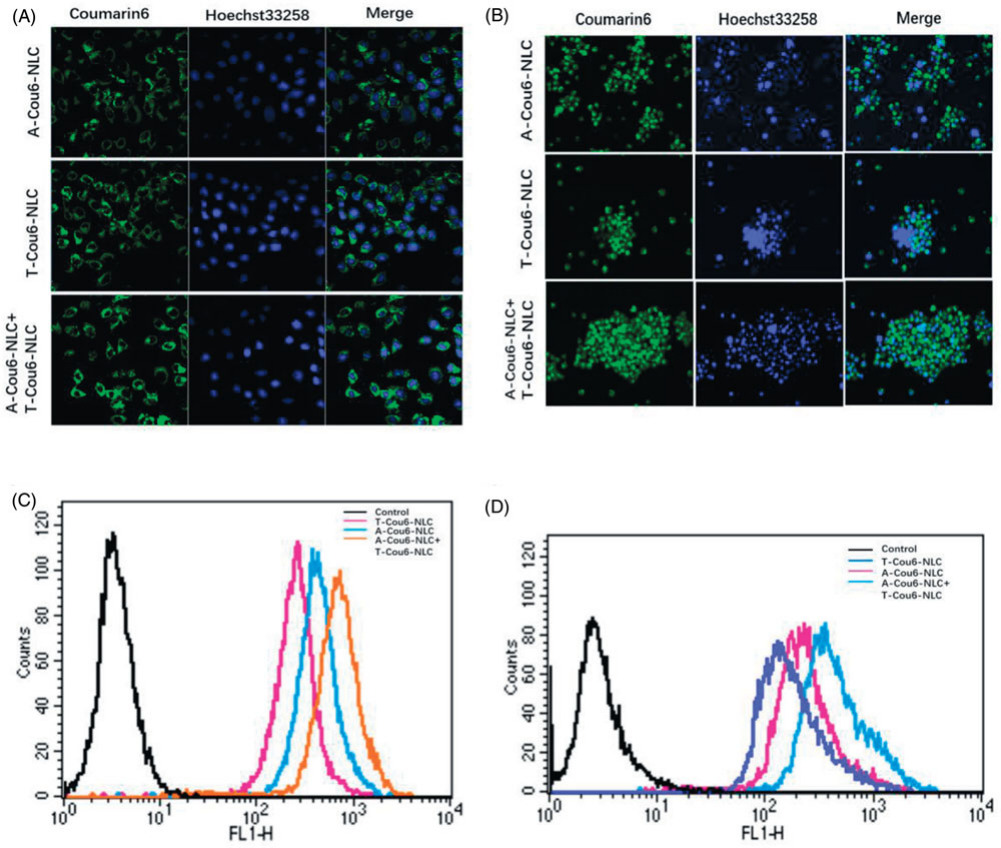
Cellular uptake of A-Cou6-NLC, T-Cou6-NLC, and combined A-Cou6-NLC + T-Cou6-NLC in NCI-H1299 cells (A) and S180 cells (B) observed by the laser confocal microscopy. Green signal represented Cou6 and blue signal represented Hoechst 33258. Quantitative cellular uptake study of A-Cou6-NLC, T-Cou6-NLC, and A-Cou6-NLC + T-Cou6-NLC in NCI-H1299 cells (C) and S180 cells (B). The result was tested by flow cytometry.

*In vitro* cellular uptake pictures of Cou6 loaded preparations by NCI-H1299 and S180 cells are shown in Figure 2(A,B). The fluorescent intensity of T-Cou6-NLC + A-Cou6-NLC was obviously stronger than single T-Cou6-NLC or A-Cou6-NLC in both NCI-H1299 and S180 cells, indicating the superior cellular uptake of the two preparations when used in combination. The results of quantitative assay of *in vitro* cellular uptake are shown in Figure 2(C,D), was in accordance with the photographs of confocal microscopy that the combined group revealed significant higher fluorescent intensity over single A-Cou6-NLC and T-Cou6-NLC. These results should be caused by the synergistic effect of the two preparations that A-Cou6-NLC actively targeted to the NCI-H1299 and S180 cells and T-Cou6-NLC targets to the CSCs at the same time.

### 3.5. *In vitro* tumor inhibition effect of the combination of AR modified NLC and TR modified NLC

**Figure 3.**
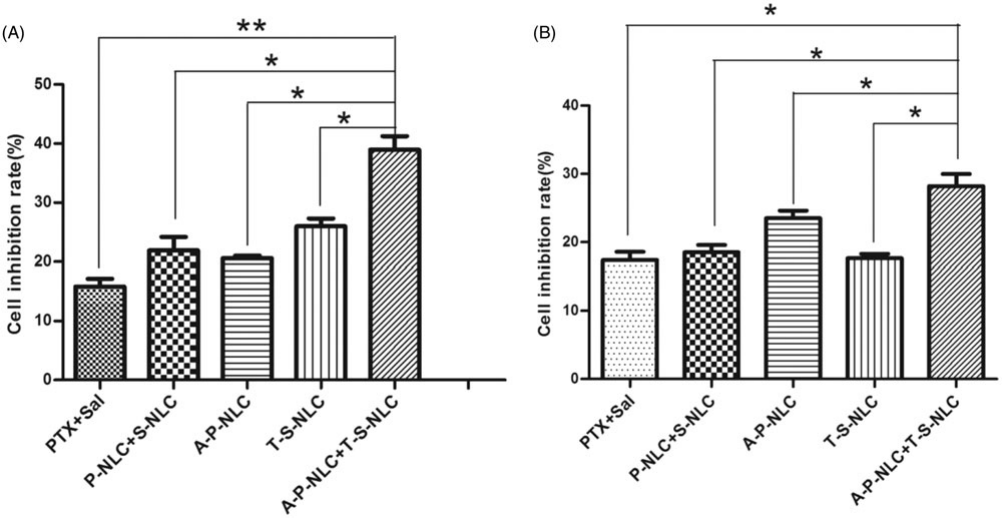
Proliferation inhibition effect of various preparations to NCI-H1299 cells (left) and S180 cells (right). Drug concentration was 0.53 μg/ml and the ratio of PTX and Sal was 1:1 (*p < .05, **p < .01).

The proliferation inhibition effect of various preparations including PTX, P-NLC, A-P-NLC, Sal, S-NLC, and T-S-NLC to NCI-H1299 and S180 cells was studied and the IC50 was calculated (Details not shown). The IC50 of the preparations to NCI-H1299 cells were as follows: PTX: 45.13 μg/ml, P-NLC: 12.21 μg/ml, A-P-NLC: 7.57 μg/ml, Sal: 28.00 μg/ml, S-NLC: 13.22 μg/ml, T-S-NLC:9.29 μg/ml. And the IC 50 of the preparations to S180 cells were: PTX: 42.40 μg/ml, P-NLC: 15.74 μg/ml, A-P-NLC: 5.94 μg/ml, Sal: 23.93 μg/ml, S-NLC: 15.01 μg/ml, and T-S-NLC: 9.43 μg/ml. It could be concluded that the IC50 of A-P-NLC and T-S-NLC was similar in both NCI-H1299 and S180 cells, thus, the concentration ratio was set to 1:1, when these two preparations were used in combination. The inhibition ratio of combined PTX + Sal, P-NLC + S-NLC and A-P-NLC + T-S-NLC to NCI-H1299 and S180 cells is shown in Figure 3, the outcomes of A-P-NLC + T-S-NLC was notably higher than the other groups in both cells. The results further confirmed that it was possible to achieve a better antitumor effect to kill cancer cells and CSCs at the same time.

### 3.6. *In vivo* tumor targeting effect of the combination of AR modified NLC and TR modified NLC

**Figure 4.**
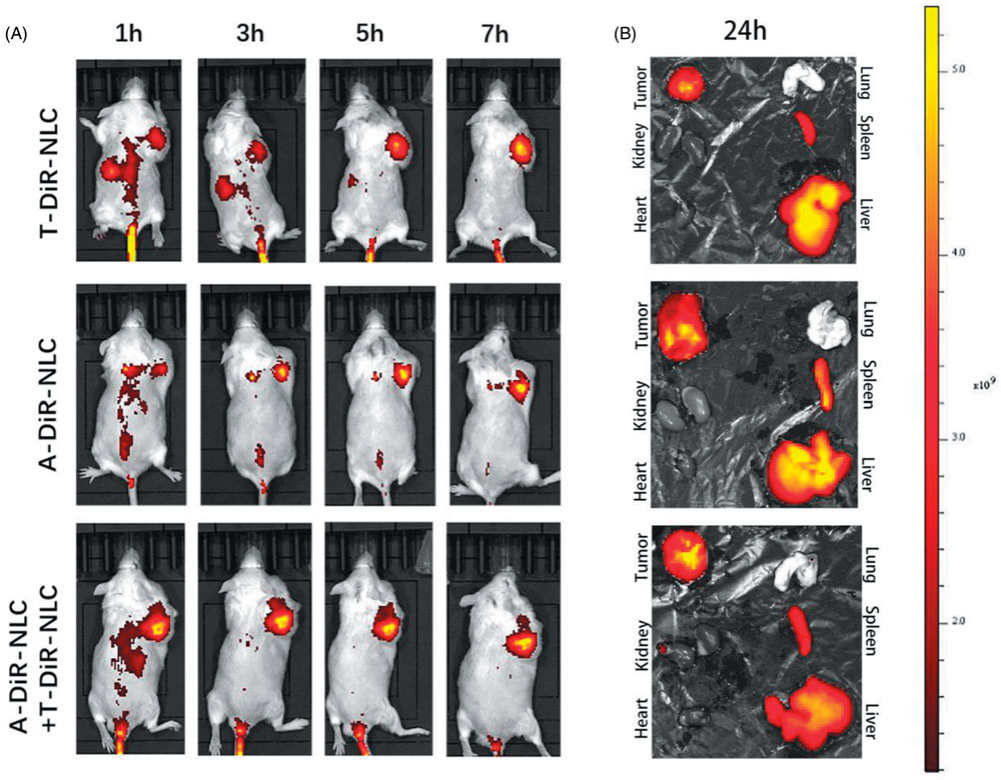
(A) *In vivo* tumor targeting effect of T-Dir-NLC, A-Dir-NLC, and combined A-Dir-NLC + T-Dir-NLC. Tumors were showed in the right armpit of the mice in the pictures. (B) *Ex vivo* fluorescent pictures of T-Dir-NLC, A-Dir-NLC, and combined A-Dir-NLC + T-Dir-NLC in tumors and normal tissues of the mice.

Dir as the *in vivo* fluorescent dye was loaded into A-NLC and T-NLC to get A-Dir-NLC and T-Dir-NLC. A-Dir-NLC, T-Dir-NLC, and A-Dir-NLC + T-Dir-NLC were injected to S180 tumor-bearing mice at the dose of 2.5 mg/kg. The *in vivo* distribution of different preparations is shown in Figure 4(A). It could be read that at the time point of 1 h, all the preparations revealed obvious tumor accumulation, and the fluorescent intensity was in the order of A-Dir-NLC +  T-Dir-NLC > A-Dir-NLC > T-Dir-NLC. As time went by, the signal became stronger in all the groups, and signal in tumor area of A-Dir-NLC + T-Dir-NLC group remained the strongest within all the time points, indicating the superior tumor targeting effect over single groups. The mice were sacrificed 24 h after the final dose, and *ex vivo* pictures of the tumors and other organs were taken (Figure 4(B)), A-Dir-NLC + T-Dir-NLC groups gave notably greater Dir intensity than A-Dir-NLC and T-Dir-NLC in tumor tissue, implying better tumor targeting effect. In a word, the *in vivo* tumor targeting assay verified the synergistic tumor targeting effect of A-Dir-NLC and T-Dir-NLC. This may be because A-Dir-NLC and T-Dir-NLC passively targeted to the tumor tissue when they passed through the leaky tumor neovascular and quickly internalized by tumor cells and CSCs, leaving enough space for the following NLC that entered the tumor tissue by EPR effect, the continuous passive targeting and active cellular uptake made the more efficient antitumor drug delivery possible.

### 3.7. *In vivo* antitumor effect of the combination of AEYLR modified NLC and TR modified NLC

**Figure 5.**
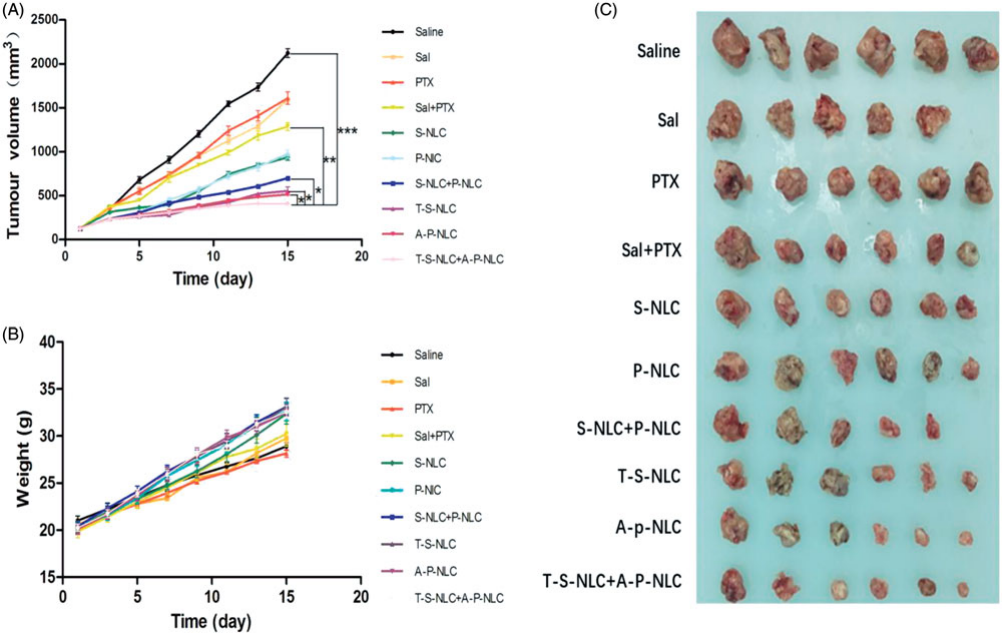
(A) Tumor volume curve in the groups that the mice were treated with different preparations. The tumor volume was documented every other day during the treatment. (*p < .05, **p < .01, ***p < .001). (B) Body weight curve in the groups that the mice were treated with different preparations. The body weight was documented every other day during the treatment. (C) The photographs of tumors in each group. The tumors were peeled off 24 h after the final dose.

The *in vivo* antitumor effect was studied using a series of preparations including Saline, PTX, Sal, PTX + NLC, S-NLC, P-NLC + S-NLC, A-P-NLC, T-S-NLC, and A-P-NLC + T-S-NLC to S180 tumor-bearing mice at the drug dose of 0.5 mg/kg. Body weight and tumor volume were recorded every other day during the treatment. According to the tumor volume curves shown in Figure 5(A), the saline group gave out the largest tumor volume at around 2200 mm^3^, representing the natural growth speed of the tumor, while all the drug groups showed tumor inhibition effect in varying extent. When used by the free form, PTX and Sal showed similar tumor suppression effect that the tumor volume in these two groups were both 1500 mm^3^. After loading to NLC, P-NLC, and S-NLC inhibited the tumor volume to about 900 mm^3^, respectively, suggesting the better inhibition effect. This was because, after loading to the drug carrier, P-NLC and S-NLC could passively enter the tumor area through EPR effect. The tumor volume of active targeting A-P-NLC and T-S-NLC groups were further decreased to around 900 mm^3^, and the value was reduced to 500 mm^3^ when we used the combined groups of A-P-NLC + T-S-NLC, suggesting the synergistic effect of the two preparations. The other combined groups of PTX + Sal and P-NLC + S-NLC also revealed better tumor suppression effect over corresponding single preparations but not so good as A-P-NLC + T-S-NLC group.

The living state of the mice was represented by the body weight in Figure 5(B), the mice body weight in all the groups increased during the treatment and the A-P-NLC, T-S-NLC, and A-P-NLC + T-S-NLC showed the highest weight, implying the better safety of these preparations. It should be mentioned that one mouse died on the 6th day in Sal group, indicating the severe adverse effect of free Sal.

The tumors were peeled off 24 h after the final treatment, weighed and used to calculate the tumor inhibition ratio. Pictures of the tumors are shown in Figure 5(C), it could be seen that the tumor volume in A-P-NLC, T-S-NLC, and A-P-NLC + T-S-NLC groups was smaller than the other groups. Tumor inhibition ratio in these groups (Table 1) especially A-P-NLC + T-S-NLC was prominently higher than the other groups.

**Table 1.** *In vivo* treatment dose, animal number, average tumor weight, and tumor inhibition ratio (IR%) of various preparations.

Group	Dose (mg/kg)	Animal number (start/end)	Average tumor weight (g)	IR(%)
Saline	–	6/6	8.17 ± 0.43	–
Sal	5	6/5	7.28 ± 0.41	10.81
PTX	5	6/6	7.52 ± 0.38	7.94
Sal + PTX	2.5 + 2.5	6/6	6.62 ± 0.66	18.96
S-NLC	5	6/6	6.41 ± 0.47	21.55
P-NlC	5	6/6	6.56 ± 0.82	19.65
S-NLC + P-NLC	2.5 + 2.5	6/5	5.97 ± 0.81	26.86#
T-S-NLC	5	6/6	5.05 ± 1.21	38.13# $
A-P-NLC	5	6/6	4.51 ± 1.07	44.76## $$
T-S-NLC + A-P-NLC	2.5 + 2.5	6/6	3.82 ± 1.19	53.24## $$&*

In comparison with Sal + PTX: #*p* < .05, ## *p* < .01; in comparison with S-NLC + P-NLC: $*p* < .05, $$*p* < .01; in comparison with T-S-NLC: &*p* < .05; in comparison with A-P-NLC: *p < .05.

To sum up, the *in vivo* antitumor study confirmed the conjecture that the combination of A-P-NLC + T-S-NLC achieved better antitumor effect while maintaining the safety.

## 4. Conclusion

In the present manuscript, we built a CD133+ specific peptide TR modified NLC (T-NLC), it's *in vitro* active tumor targeting effect was validated using Cou6 as the fluorescent probe, it's *in vitro* tumor inhibition effect was confirmed using Sal as the model drug. After that, T-NLC was used in combination with A-NLC (EGFR specific small peptide AR modified NLC), the combined T-NLC and A-NLC exhibited more tumor cell accumulation both *in vitro* and *in vivo*. The *in vivo* antitumor assay further demonstrated the efficiency and safety of the combination of PTX loaded A-P-NLC and Sal loaded T-S-NLC.
